# Chylothorax Following Endoscopic Ultrasound‐Guided Fine‐Needle Aspiration: A Rare Complication and Its Anatomical Risk Factors

**DOI:** 10.1002/rcr2.70732

**Published:** 2026-08-19

**Authors:** Saima Bandarkar, Ahmed Abdelsamad, Yousef Aleid, Ahmed Nasr, Talal AlSaeed, Essa AlGunaim, Derar AlShehab, Adel Ayed, Mohammed I. Ghanbar, Sulaiman Khadadah

**Affiliations:** ^1^ School of Medicine Royal College of Surgeons in Ireland – Medical University of Bahrain Muharraq Bahrain; ^2^ Interventional Pulmonary Unit Chest Disease Hospital Kuwait City Kuwait; ^3^ Chest Diseases Department, Faculty of Medicine Ain Shams University Cairo Egypt; ^4^ Thoracic and Foregut Surgery Department Chest Disease Hospital Kuwait City Kuwait; ^5^ Radiology Department Chest Disease Hospital Kuwait City Kuwait; ^6^ College of Medicine and Health Sciences Abdullah Al Salem University Kuwait City Kuwait; ^7^ Johns Hopkins University Baltimore Maryland USA

**Keywords:** chylothorax, endoscopic ultrasound‐guided fine‐needle aspiration, pleural effusion, thoracic duct injury, thoracic lesions

## Abstract

Chylothorax is an exceptionally rare complication of endoscopic ultrasound‐guided fine‐needle aspiration (EUS‐FNA), with only a limited number of cases reported in the literature. We present the case of a 66‐year‐old man who presented with dyspnoea 3 days after EUS‐FNA of a paraoesophageal left upper lobe lung mass. Chest radiography and CT demonstrated a left‐sided pleural effusion, and image‐guided pleural drainage yielded milky fluid whose analysis confirmed chylothorax. He was managed conservatively with a 14‐French intercostal chest drain (ICD), dietary modification and octreotide therapy, achieving complete clinical recovery. This case underscores the delayed and potentially under‐recognised presentation of post‐EUS‐FNA chylothorax and raises the possibility that anatomical relationships between thoracic lesions and the thoracic duct may represent a potential risk factor for this uncommon complication.

## Graphical abstract

This case describes the successful conservative management of chylothorax, a rare complication following EUS‐FNA. A low‐output leak of 260 mL/day resolved fully within days using intercostal chest drainage, dietary modification and octreotide therapy. The consistent left‐sided lesion pattern across reported cases raises a possible anatomical risk factor, though this cannot be confirmed from a single case.

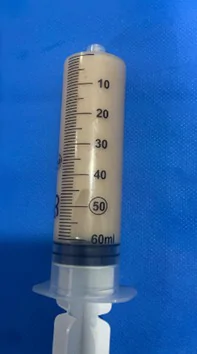

## 1 Introduction

Chylothorax is defined as an abnormal accumulation of chyle, a lymphatic fluid carried by the thoracic duct, within the pleural space surrounding the lungs [1, 2, 3]. Traumatic chylothorax is an exceptional complication following thoracic procedures that result in injury to the thoracic duct or its tributaries [4]. Surgery and trauma are among the most common causes of traumatic chylothorax, with the main presenting symptom being shortness of breath [2, 5]. In cases of nontraumatic chylothorax, malignancies such as lymphomas are strongly associated with this complication [1]. Overall, the incidence of traumatic chylothorax has increased due to the growing number of thoracic interventions, accounting for over 50% of cases reported [5]. EUS‐FNA is a widely performed, minimally invasive procedure with low reported complication rates (as low as 0.7%) [6, 7]; nonetheless, unexpected complications such as thoracic duct injury can still occur, as documented in only a handful of published cases [8, 9]. First‐line management is typically conservative, involving the implementation of a fat‐free diet and octreotide therapy, with ICD insertion reserved for cases with large pleural effusions [10, 11]. Failure of conservative management to achieve resolution would necessitate surgical intervention, including thoracic duct ligation via video‐assisted thoracoscopic surgery (VATS) or thoracotomy [11]. We present a rare case of left‐sided chylothorax secondary to thoracic duct injury following EUS‐FNA and highlight the importance of early diagnosis and multidisciplinary collaboration to ensure timely management of this uncommon complication [2].

## 2 Case Report

**FIGURE 1:**
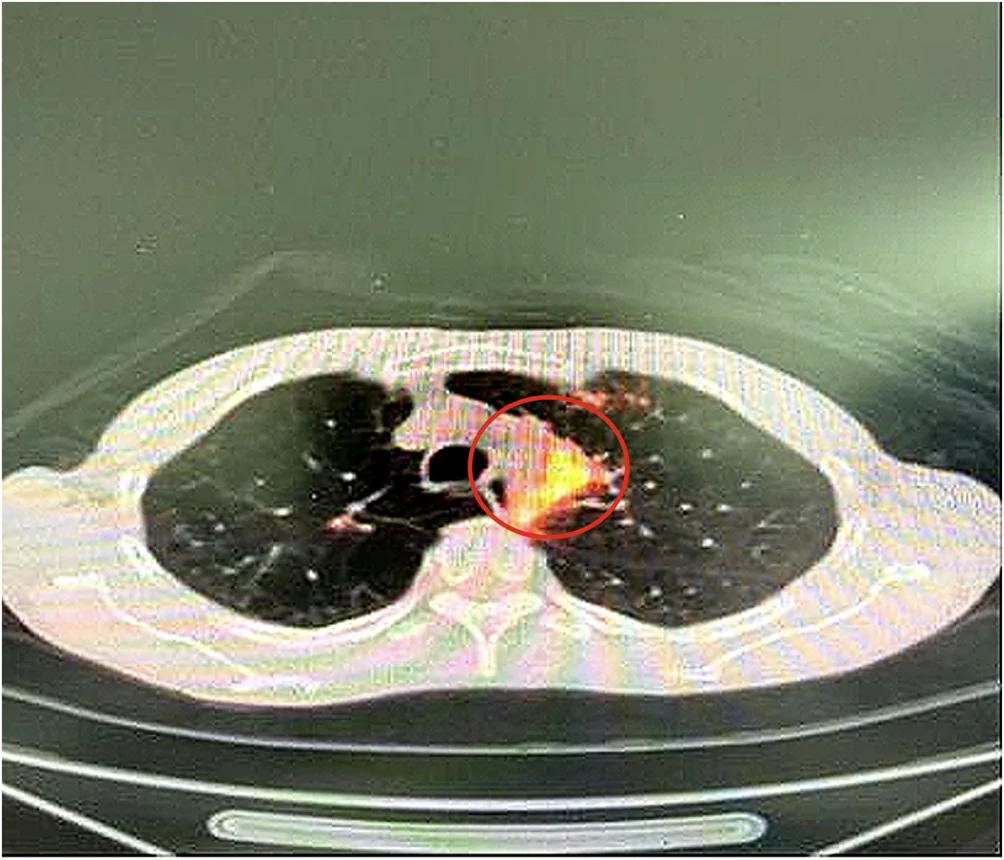
PET‐CT demonstrating intense FDG uptake within the left upper lobe, consistent with a hypermetabolic pulmonary mass (SUVmax: 13.7).

**FIGURE 2:**
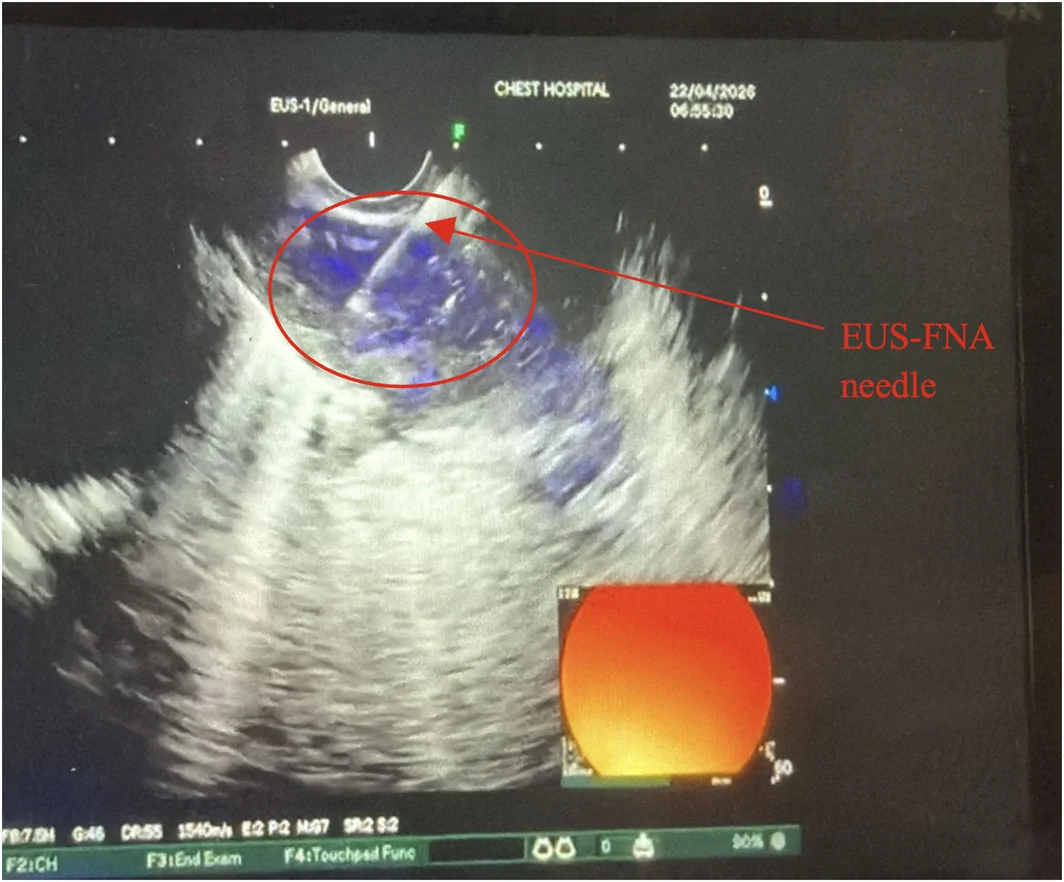
Endoscopic ultrasound‐guided fine‐needle aspiration (EUS‐FNA) of a heterogeneous paraoesophageal left upper lobe lung mass.

A 66‐year‐old male patient was referred to the hospital with cough, shortness of breath and a known history of COPD. Chest CT revealed a 60 × 34 × 53 mm spiculated solid lesion in the left upper lobe, demonstrating a positive bronchus sign. He had previously undergone bronchoscopy at a secondary hospital, which showed no endobronchial lesions, and bronchoalveolar lavage returned negative for infection with benign cytology. Further evaluation with PET‐CT showed intense hypermetabolic activity in the left upper lobe lesion with a maximum standardised uptake value (SUVmax) of 13.7 (Figure 1). Following a thorough multidisciplinary team (MDT) discussion, the patient underwent an endoscopic ultrasound‐guided fine‐needle aspiration (EUS‐FNA) of a paraoesophageal left upper lobe lung mass (Figure 2). Preliminary rapid on‐site evaluation (ROSE) of the specimen showed necrotising granuloma, which was further established by a subsequent cytopathological report. He was discharged home the following day with an unremarkable CXR and negative tuberculosis GeneXpert results.

**FIGURE 3:**
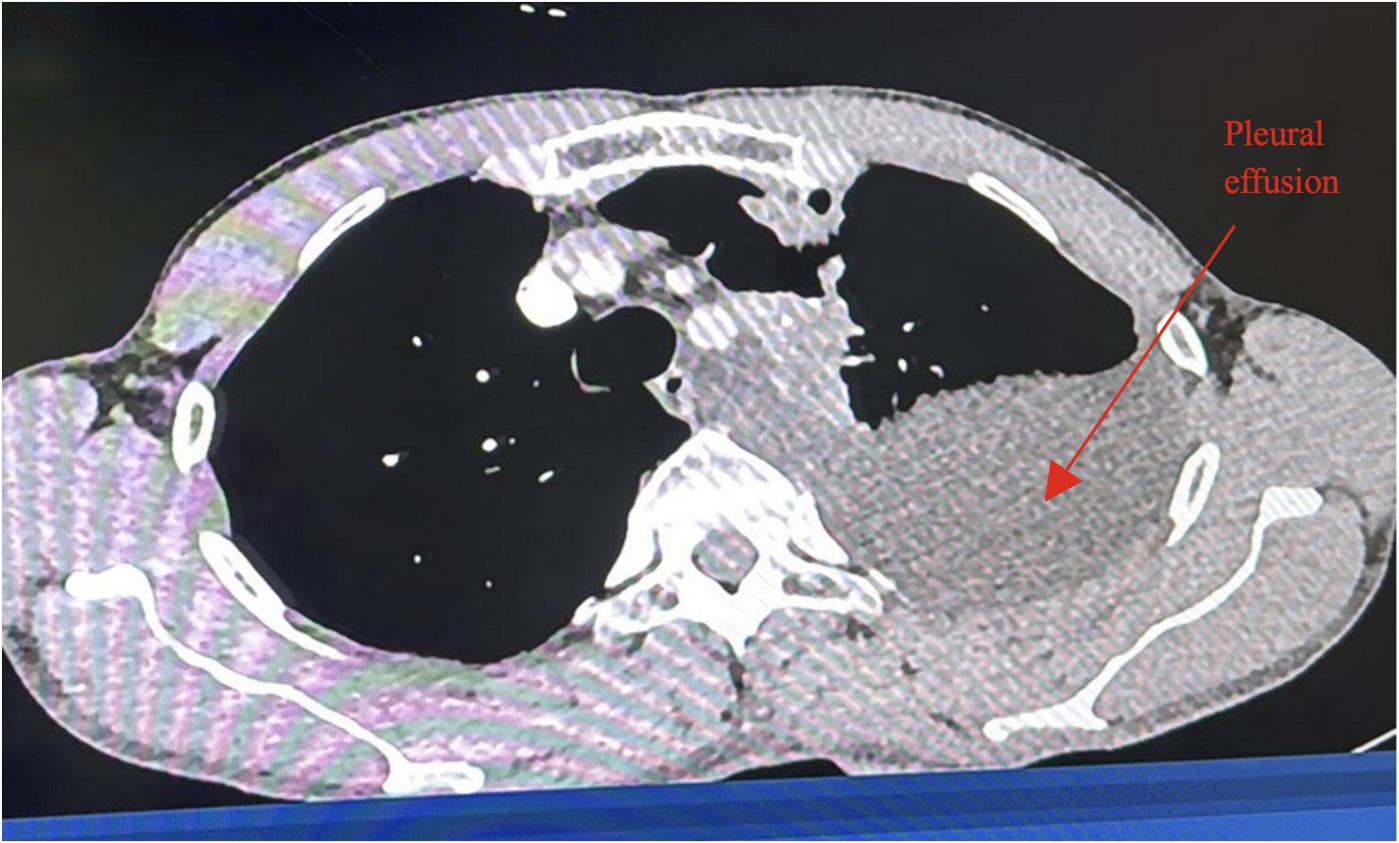
CT image following EUS‐FNA demonstrating a moderate to large left‐sided pleural effusion (secondary to chylothorax).

**FIGURE 4:**
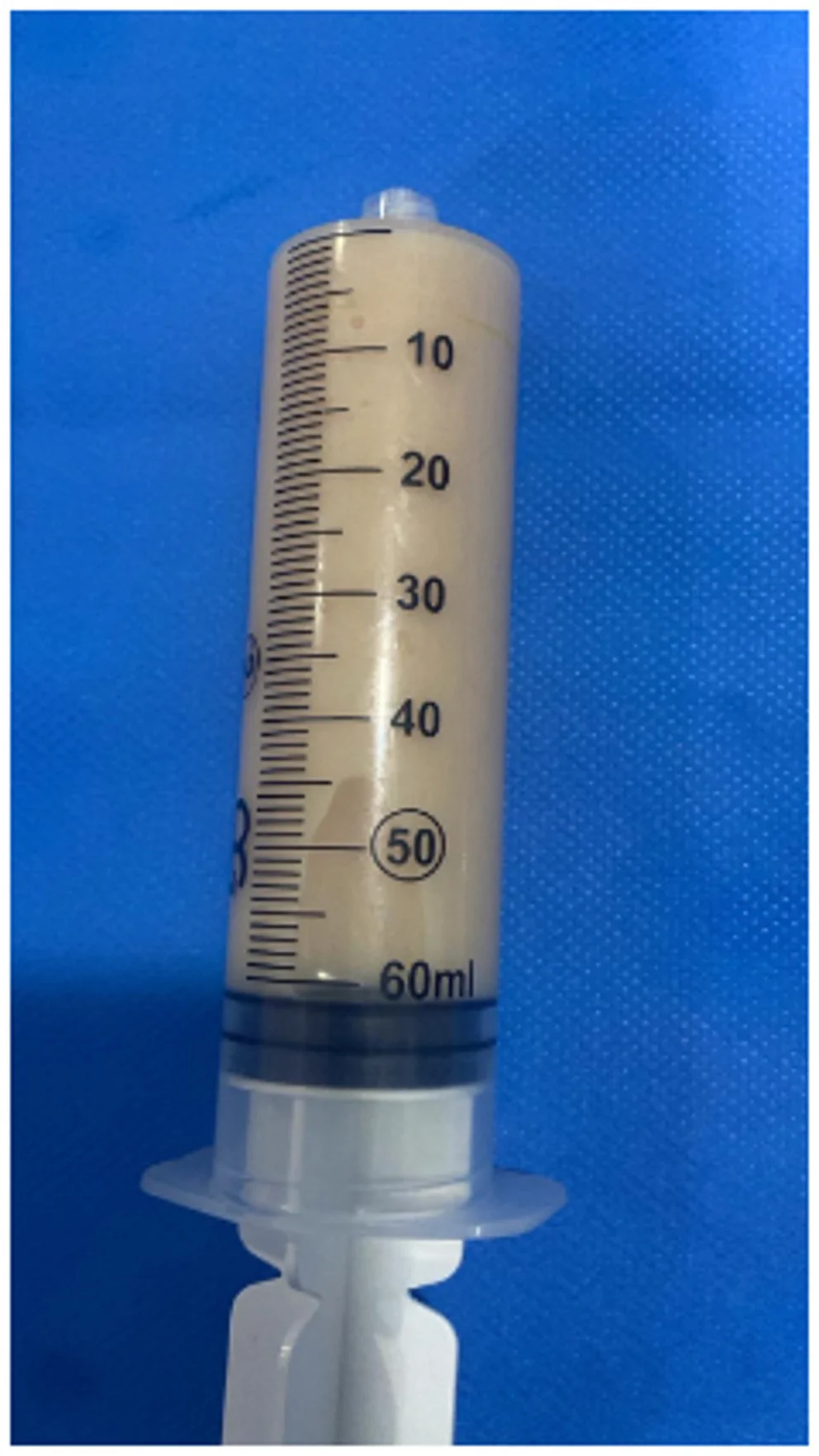
Gross appearance of the aspirated pleural fluid, demonstrating its characteristic milky, chylous quality.

Three days following EUS‐FNA, the patient returned to the hospital with complaints of dyspnoea and chest pain. Left‐sided pleural effusion was evident on chest radiography and CT (Figure 3), prompting image‐guided insertion of a 14‐French intercostal chest drain, which drained milky pleural fluid (Figure 4). The diagnosis of chylothorax was established based on the characteristic milky‐white appearance of the pleural fluid alongside biochemical analysis demonstrating a markedly elevated pleural fluid triglyceride level of 24.67 mmol/L and the presence of chylomicrons. At presentation, he was haemodynamically stable and was managed with intravenous fluids while remaining nil per os (NPO). A low‐fat diet supplemented with medium‐chain triglycerides was commenced, alongside administration of octreotide 50 micrograms three times daily subcutaneously. Conservative management was continued, resulting in a marked reduction in pleural drainage from 260 mL to < 5 mL over 4 days. He was closely monitored for the next week, adequately tolerating the low‐fat diet, with stable vital signs and no complaints of pain or shortness of breath. Daily chest radiographs demonstrated complete re‐expansion of the left lung and resolution of the pleural effusion. A dietary challenge with full‐cream milk was successfully tolerated and the patient was subsequently discharged in good condition. On subsequent follow‐up, mycobacterial cultures from the biopsy specimen grew *Mycobacterium avium* complex (MAC), in contrast to the earlier negative bronchoalveolar lavage cultures; the patient was commenced on treatment for MAC pulmonary disease and was improving at follow‐up.

## 3 Discussion

EUS‐FNA is widely regarded as a safe and minimally invasive diagnostic procedure with studies showing complication rates as low as 0.7% [6]. Although adverse effects such as bleeding, perforation and infection have been previously reported [7], thoracic duct injury resulting in chylothorax remains a rare occurrence. To our knowledge, this represents only the third reported case of chylothorax following EUS‐FNA, highlighting the need for greater awareness of potential risk factors and timely management of this uncommon complication.

Diagnosis of chylothorax is confirmed biochemically and microscopically on pleural fluid analysis. A pleural fluid triglyceride level of > 1.24 mmol/L with a cholesterol level of < 5.18 mmol/L is diagnostic of chylothorax [3]. In instances where the triglyceride level falls between the thresholds for pseudochylothorax and chylothorax (0.56–1.24 mmol/L), lipoprotein analysis is required to confirm the diagnosis by demonstrating the presence of chylomicrons [3]. In our patient, the markedly elevated triglyceride level of 24.67 mmol/L alongside confirmed chylomicrons satisfied both these criteria unequivocally, thus supporting the gross appearance seen on drainage. The mechanism of thoracic duct injury is likely multifactorial. Due to anatomical variation across patients, the relationship of the thoracic duct to its neighbouring mediastinal structures may not always be clearly appreciated during EUS‐FNA, particularly given the limited visualisation with respect to the unavoidable blind zone [12]. In the first reported case of chylothorax secondary to EUS‐FNA, distortion of the normal lymphatic network by a right‐sided aortic arch was hypothesised as a contributing factor [8]. Interestingly, all reported cases of post‐EUS‐FNA chylothorax identified by our literature review have involved left‐sided thoracic lesions, which raises the possibility that anatomical relationships between left‐sided thoracic targets and the thoracic duct may represent a potential, though still under‐recognised, risk factor for lymphatic injury during EUS‐FNA [8, 9]. Anatomically, the thoracic duct crosses from right to left at the level of T5‐T6, passing behind the oesophagus before ascending on the left side of the posterior mediastinum [12]. A transoesophageal needle directed towards a left‐sided paraoesophageal target therefore traverses the region most likely to contain the duct or its tributaries. However, with only three reported cases and differing proposed mechanisms, including the right‐sided aortic arch described in the first case, these observations remain hypothesis‐generating rather than established. Furthermore, procedural factors such as lesion proximity to lymphatic structures, needle trajectory, penetration depth and repeated needle passes may also theoretically contribute to the risk of lymphatic injury, although these specific risk factors have not clearly been established [7, 13].

Effective management requires a multidisciplinary approach with collaboration between pulmonology, thoracic surgery, radiology and nutrition being essential for early diagnosis [2]. Conservative management remains the cornerstone of management, involving pleural drainage with ICD insertion, dietary modification with medium‐chain triglycerides and pharmacological therapy such as octreotide [10, 11]. However, persistent high‐output leaks may require further surgical intervention; in our patient, conservative management was sufficient to allow for complete clinical recovery [11]. Reviewing the management of the two previously reported cases provides useful context for understanding the significance of our management plan. In the first reported case, the patient had a massive output of 1800 mL 2 days after EUS‐FNA of a left mediastinal mass; conservative management was not sufficient, and surgical intervention was necessary via a left mini‐thoracotomy [8]. Similarly, a left‐sided thoracotomy and talc pleurodesis were indicated for the patient in the second case, with a high output of 500 mL and a persistent pleural effusion [9]. In comparison, our patient had a low‐output leak (peak drainage 260 mL/day) that resolved completely with conservative measures alone. This contrast suggests that drainage volume is an important determinant of whether conservative management succeeds, and that low‐output post‐EUS‐FNA chylothorax, particularly when recognised early, may be managed without surgery.

The rarity of post‐EUS‐FNA chylothorax limits clinical familiarity with the condition and contributes to challenges in early recognition and management. The novel nature of chylothorax as a complication and its delayed presentation both make diagnosis challenging and call for meticulous attention [8, 9]. It is therefore important for clinicians to maintain a high index of suspicion when new pleural effusions develop following EUS‐guided procedures, particularly when the drainage is milky or triglyceride are elevated [1]. Additionally, because the thoracic duct is not directly visualised at EUS, careful procedural planning informed by awareness of its typical left‐sided mediastinal course may help reduce the risk of this uncommon yet potentially significant complication [8].

In conclusion, chylothorax is a rare but recognisable complication of EUS‐FNA that may present with a delay of several days after an apparently uneventful procedure. In contrast to the two previously reported cases, both of which required surgical intervention, our patient had a low‐output leak that resolved completely with conservative management, suggesting that early recognition and low drainage volume may identify patients in whom conservative management is more likely to succeed. All three reported cases to date have involved left‐sided targets, which is consistent with the course of the thoracic duct behind the oesophagus and along the left posterior mediastinum. Although too few cases exist to establish precise risk, clinicians should remain vigilant about this anatomical relationship and complication when sampling left‐sided paraoesophageal lesions.

## Author Contributions

Saima Bandarkar prepared the first draft of the manuscript and contributed to its review. Yousef Aleid and Sulaiman Khadadah were the primary physicians responsible for the patient's care, including the diagnostic procedure, and Ahmed Nasr performed the image‐guided pleural drainage. Ahmed Abdelsamad, Ahmed Nasr, Talal AlSaeed, Essa AlGunaim, Derar AlShehab, Adel Ayed and Mohammed I. Ghanbar contributed to manuscript review, editing and critical revision for important intellectual content. All authors approved the final version of the manuscript.

## Consent

The authors declare that written informed consent was obtained for the publication of this manuscript and accompanying images and attest that the form used to obtain consent from the patient complies with the journal's requirements as outlined in the author guidelines.

## Conflicts of Interest

The authors declare no conflicts of interest.

## Data Availability

Data sharing not applicable to this article as no datasets were generated or analysed during the current study.
